# Fluorine-Containing Drug Administration in Rats Results in Fluorination of Selected Proteins in Liver and Brain Tissue

**DOI:** 10.3390/ijms23084202

**Published:** 2022-04-11

**Authors:** Andrzej Gawor, Zdzislaw Gajewski, Leszek Paczek, Bozena Czarkowska-Paczek, Anna Konopka, Grzegorz Wryk, Ewa Bulska

**Affiliations:** 1 Biological and Chemical Research Centre, Faculty of Chemistry, University of Warsaw, Zwirki i Wigury 101, 02-089 Warsaw, Poland; agawor@chem.uw.edu.pl (A.G.); a.konopka@cnbc.uw.edu.pl (A.K.); gwryk@cnbc.uw.edu.pl (G.W.); ebulska@chem.uw.edu.pl (E.B.); 2 Center for Translational Medicine, Warsaw University of Life Science, Nowoursynowska 100, 02-797 Warsaw, Poland; zgajewski@supermedia.pl; 3 Department of Immunology, Transplantology and Internal Diseases, Medical University of Warsaw, Nowogrodzka 59, 02-006 Warsaw, Poland; leszek.paczek@wum.edu.pl; 4 Department of Bioinformatics, Institute of Biochemistry and Biophysics, Polish Academy of Sciences, Pawinskiego 5a, 02-106 Warsaw, Poland; 5 Department of Clinical Nursing, Medical University of Warsaw, Ciolka Street 27, 01-445 Warsaw, Poland

**Keywords:** fluorinated drug, protein fluorination, drug biotransformation, fluorine

## Abstract

In many pharmaceuticals, a hydrogen atom or hydroxyl group is replaced by a fluorine to increase bioavailability and biostability. The fate of fluorine released from fluorine-containing drugs is not well investigated. The aim of this study was to examine possible fluorination of proteins in rat liver and brain after administration of the fluorinated drug cinacalcet. We assigned 18 Wistar rats to a control group (*n* = 6) and a group treated with cinacalcet (2 mg kg^−1^/body weight, 5 days/week), divided into 7 day (*n* = 6) and 21 day (*n* = 6) treatment subgroups. Fluorinated proteins were identified using a free proteomics approach; chromatographic separation and analysis by high-resolution mass spectrometry; peptide/protein identification using the Mascot search algorithm; manual verification of an experimentally generated MS/MS spectrum with the theoretical MS/MS spectrum of identified fluorinated peptides. Three fluorinated proteins (spectrin beta chain; carbamoyl-phosphate synthase [ammonia], mitochondrial; 6-phosphofructo-2-kinase/fructose-2, 6-bisphosphatase 1) were identified in the liver and four (spectrin beta chain, dihydropyrimidinase-related protein 4, prominin-2, dihydropyrimidinase-related protein 4) in the brain tissue after 21 days of cinacalcet treatment, but not in the control group. Introduction of fluorine into an organism by administration of fluorinated drugs results in tissue-specific fluorination of proteins.

## 1. Introduction

Fluorine is widely used in industry for the synthesis of various particles, especially in the pharmaceutical sector. In more than 25% of all pharmaceuticals, a hydrogen atom or hydroxyl group is replaced chemically by a fluorine (-F) or trifluoromethyl group (-CF3) during drug development. The characteristics of fluorine include high electron affinity, lipophilicity, and bioavailability, which extends the half-life of the drug and increases its potency [1,2,3]. Fluorinated drugs are used for immunological diseases, hyperlipemia, malaria, hyperparathyroidism, psychiatric disorders, peptic ulcer disorders, selected tumors, and bacterial, viral, or fungal infections, among other disease targets [1,4,5]. The applications of fluorine for drug design have expanded to include new fluorinated motifs [6,7]. The benefits of therapy with fluorinated drugs are well known, but much less is known about potential threats and risks. Adverse effects of these drugs are well documented, and appropriate information is included in the summary of product characteristics (SmPCs) [8], although in many cases, the mechanisms are not fully defined [9]. Investigations should take into account the effect of increased fluorine intake on physiology and how metabolites of fluorine-containing particles might influence biochemical pathways. Drug metabolism plays a crucial role in drug-associated health outcomes. The biotransformation of fluorinated compounds is linked to the departure of fluoride as a leaving group. Such defluorination can occur spontaneously or during metabolic reactions if the molecule is sufficiently electrophilic to react directly with nucleophilic groups in proteins. Examples of such groups are the amino group in lysine and the hydroxyl group in serine, resulting in the formation of fluorine ion or trifluoroacetic acid. In rare cases, spontaneous defluorination of the -CF3 group may occur [5,10].

The outcomes of drug defluorination are not completely clear, but investigations into the metabolism of fluorinated molecules have revealed that these metabolic pathways can entail several consecutive steps culminating in fluorinated toxin release [7,11]. The question arises of whether defluorination could result in subsequent fluorination of proteins in the organism, with positive or negative consequences. Thanks to its biochemical and physical properties, fluorine easily penetrates the cell membranes of hard and soft tissues, and the fluid and tissue concentration of fluorine is not under homeostatic control [12]. The organs most vulnerable to the harmful effects of fluorine are liver and kidneys, but progressive degeneration also can be seen with exposure in the small intestine, colon, brain, spine, and heart [13,14,15]. For these reasons, the identification and determination of fluorine-containing molecules in tissues would aid understanding of the fate of fluorine after fluorine-containing drug administration, especially regarding interaction among proteins, ionic fluorides, and covalently bound fluorine in the drugs. The aim of this study was to examine possible fluorination of proteins in rat liver and brain after systemic administration of the fluorinated drug cinacalcet.

## 2. Results

**Figure 1:**
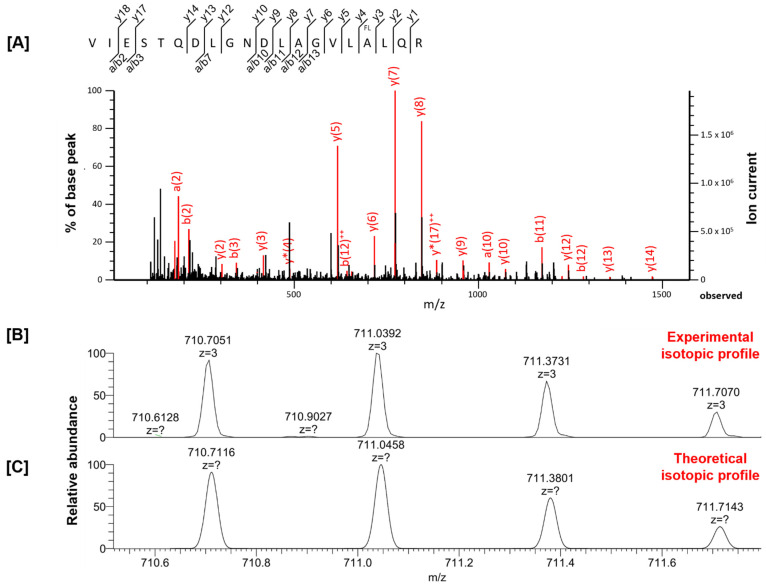
(**A**) Evaluation of MS/MS spectrum for the peptide VIESTQDLGNDLAGVLALQR from the spectrin beta chain, obtained from a brain tissue sample after 21 days of cinacalcet administration. (**B**) Experimental isotopic profile for the peptide VIESTQDLGNDLAGVLALQR. (**C**) Theoretical isotopic profile for the peptide VIESTQDLGNDLAGVLALQR simulated in silico (resolving power: 26,000 FWHM).

The MS-based proteomics approach was successfully applied to identify proteins in the examined tissues [16]. The resulting MS/MS data were used in an automated search with the Mascot algorithm. During the sequence alignment, the experimentally generated MS/MS spectrum was compared to the theoretical MS/MS spectrum of each peptide in the database, and a score, representing the degree of correlation, was calculated for each peptide. Using this method, 2000–3500 (depending on the examined tissue) proteins were identified with at least one unique peptide, including several peptides with fluorine substitutions. The main problem associated with automated search algorithms for protein identification is false-positive hits from random matching of experimental and theoretical data [17,18]. To eliminate false positives, we decided to critically evaluate the Mascot data by manual inspection of MS/MS spectra. Only 7 peptides proposed by the Mascot algorithm were confirmed as fluorinated after manual inspection of the raw MS data. We summarized the proteins with fluorine substitutions after 21 days of cinacalcet treatment (Table 1). The position of fluorination of alanine (A) and fluorination of phenylalanine (F) were indicated in bold red. The results presented in Table 1 include the parameters of database searches with the Mascot results for each peptide. We did not confirm any fluorinated proteins after 7 day treatment. It is worth highlighting that all fluorine-containing proteins identified and further manually revised were not identified as fluorinated in the control group. The MS/MS spectra as .mgf file for all fluorinated peptides were located in Supplementary Material. Figure 1 gives an example of manual inspection of the presence of fluorine in proteins. It shows the MS/MS spectrum for the peptide VIESTQDLGNDLAGVLALQR from the spectrin beta chain, which was obtained from a brain sample after a 21-day administration of cinacalcet. 

## 3. Discussion

To the best of our knowledge, this study is the first to show that administration of fluorine-containing drugs could result in protein fluorination in mammals. By examining rat liver and brain tissue after 21 days of cinacalcet administration, we identified one fluorinated protein (spectrin beta chain) common to both tissues, plus two other fluorinated proteins in liver and three in brain that did not overlap between the tissues. These proteins were not fluorinated in the same tissues in control rats. We did not observe protein fluorination after 7 days of cinacalcet treatment. This could be a result of the pharmacokinetics of cinacalcet. Its initial half-life is approximately 6 h, the terminal half-life ranges from 30 to 40 h, and steady state is achieved before 7 days [19]. Because fluorinated amino acids are not generally recognized by the endogenous protein synthesis apparatus, the effect of fluorine-containing drugs we detect here implicates direct fluorination of the proteins themselves and not at some stage of their synthesis [20]. From among the fluorine-containing drugs, we chose cinacalcet, a type II calcimimetic and allosteric activator of CaR (calcium-sensing receptor) that changes the receptor’s structural conformation to increase sensitivity to eCa^2+^ cinacalcet shifts the Ca–parathyroid hormone concentration-response curve to the left and leads to a dose-dependent decrease in parathyroid hormone secretion [9]. We selected this drug because it contains fluorine in the form of one -CF3 group and treatment with it is chronic, so that patients experience a lengthy exposure. Investigations of cinacalcet metabolism did not point to possible defluorination, regardless that despite the strength of C–F bonds, there are many examples of its rupture during metabolism [7,21].

The effect of fluorination on protein properties has been studied for years and derives mainly from the electronegativity of fluorine. The C–F bond is polarized in the opposite direction from the C–H bond and is more stable and less polarized, resulting in greater hydrophobicity [22,23]. Fluorination changes hydration-free energy by altering side chain–water interactions and the number of backbone–water hydrogen bonds via changes in the relative side-chain–backbone conformation [23]. The increased protein hydrophobicity is not generally accompanied by shape changes. Although fluorinated residues are larger than the hydrocarbon side chains that they replace, they closely match their shape [20,24].

The main effect of fluorination of protein particles is increased stability, largely because of the increased hydrophobicity, but by various mechanisms. Fluorination changes the folding rate by decreasing unfolding and increasing folding, as the hydrophobic effect is the main driving force in protein folding [22,25]. Fluorination also results in increased thermal and proteolytic stability of proteins, and resistance to proteolysis reflects a slower rate of unfolding rather than an inability of proteases to act on fluorinated substrates [22,25]. Fluorination can modulate folding energy by lengthening or shortening binding and interaction potentials, generating unbinding pathways, or mechanically stabilizing adjacent non-fluorinated domains [26].

Fluorinated proteins have similar properties that result in specific protein–protein interactions. Peptides may self-segregate by fluorination and non-fluorination [24,27], but some data reveal that single fluorine labeling of phenylalanine or tryptophan does not change a protein’s thermodynamic stability or folding kinetics [28]. Additionally, the properties of fluorinated protein particles depend on where the fluorinated amino acid is and the location of fluorine in the side chain [29].

Data regarding the biological activity of fluorinated proteins are controversial. Some results suggest that fluorination could improve stability without influencing biological activity [22,30], whereas others have reached contrasting conclusions. Fluorination may increase ligand affinity [31] or enzyme activity [32], but studies show that protein fluorination is not fully predictable or generalizable and could depend on the fluorine source and environmental conditions. For these reasons, examining fluorinated proteins in specific tissues after exposure to fluorine-containing drugs is of great value and expands the investigational landscape regarding biotransformation and the fate of fluorine in the organism.

In the current work, spectrin beta chain was the fluorinated protein found in both investigated tissues. According to UniProtKB [33], spectrin is involved in building cytoskeleton and serves as a binding protein for actin, ankyrin, cadherin, calmodulin, GTPase, phospholipids, and RNA. Its biological function is related to plasma membrane organization together with regulation of membrane protein localization. Spectrin also is involved in mediation of transport from the Golgi to the plasma membrane and the endoplasmic reticulum to Golgi vesicles, axon guidance, the MAPK cascade, mitotic cytokinesis, and positive regulation of interleukin-2 production. Carbamoyl-phosphate synthase [ammonia], mitochondrial, an enzyme identified in liver tissue and in general involved in the urea cycle, plays an important role in removing excess ammonia from the cell. This protein takes part in glutamine metabolic processes, hepatocyte differentiation, liver development, homocysteine metabolism, synthesis of pyrimidine nucleobase, and cellular response to cAMP, fibroblast growth factor, glucagon stimulus, and others [33]. Another fluorinated protein in the liver tissue, 6-phosphofructo-2-kinase/fructose-2, 6-bisphosphatase 1, in its native form takes part in the fructose 2,6-bisphosphate metabolic process [33]. Additional fluorinated proteins identified in brain tissue were dihydropyrimidinase-related protein 4, which exerts hydrolase activity on carbon–nitrogen bonds and is involved in nervous system development and neuron death; tRNA phosphotransferase 1, which is involved in regulation of protein kinase activity and tRNA processing and splicing; and prominin-2, which regulates endocytosis and pinocytosis, cell projection organization, protein phosphorylation, and GTPase activity. Considering these observations, we could hypothesize that the stability and possibly function of fluorinated proteins in liver and brain could be altered compared to their native counterparts, raising the question of clinical consequences. Based on our results and data in the literature, we cannot speculate on whether possible changes would be beneficial or harmful over the short or long term [34]. Nevertheless, some protein features change, which could explain some adverse reactions listed in the SmPC for cinacalcet, such as vertigo, headache, anorexia, paresthesia, and nausea and vomiting, as the mechanisms for these remain to be clarified [9]. We note that our experiment was conducted in healthy animals, and these drugs are administered for disease conditions in humans. These different organismal environments could influence protein fluorination and its consequences.

In summary, fluorine is widely used, especially in the pharmaceutical industry to increase drug stability and bioavailability, and in this way is introduced into organisms. Our current results clearly indicate that administration of fluorinated drugs results in accumulation of fluorine and in protein fluorination that is tissue specific in the organism. It was revealed after 21 days of treatment, but not after 7 days. Therefore, fluorinated drugs that are administered briefly (e.g., antibiotics) may not cause protein fluorination, and the pattern of fluorination may depend on the individual drug and also on the clinical condition of the patient. The outcomes of these changes, whether beneficial, harmful, or neutral, are unknown. Thus, the organismal fate of fluorine in medications and the potential biochemical and clinical consequences should be examined in clinical trials of these drugs, especially now that appropriate methodology is available [15]. The resulting information should be incorporated into the SmPC.

## 4. Study Limitations

We only evaluated one drug as a source of fluorine, and other drugs could fluorinate different proteins in different tissues. We also did not check the biological activity of fluorinated proteins in health and disease and did not compare their activity to that of native proteins. However, this study is the first to show fluorination of proteins in the organism after drug administration, and further studies of this phenomenon are required.

## 5. Materials and Methods

**Figure 2:**
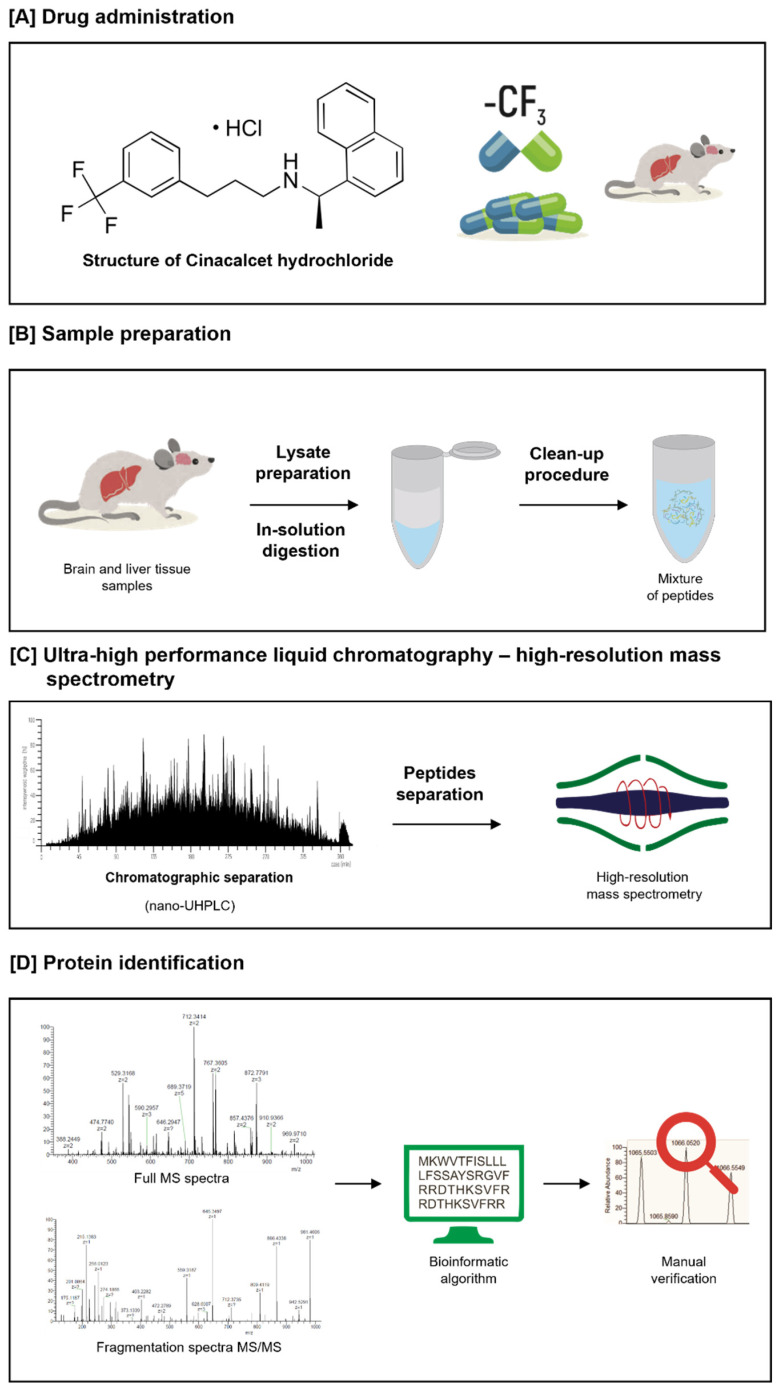
Experimental proteomics workflow for identification of proteins and verification of the presence of fluoridated peptides: (**A**) Cinacalcet drug administration; (**B**) Sample preparation to proteomic analysis; (**C**) Chromatographic peptides separation by ultra-high liquid chromatography (nano-UHPLC) and analysis by high-resolution mass spectrometry; (**D**) Data analysis including peptide/protein identification with the Mascot search algorithm and manual inspection of an experimentally generated MS/MS spectrum with the theoretical MS/MS spectrum of identified fluorinated peptides.

Identifying fluorination of liver and brain proteins after administration of fluorine-containing drugs relies on a label-free proteomics approach, with the following steps: (i) sample preparation, including protein extraction, reduction, alkylation and in-solution digestion; (ii) chromatographic separation by liquid chromatography (nano-UHPLC) and analysis by high-resolution mass spectrometry (MS; Orbitrap; Thermo Scientific, Bartlesville, OK, USA); (iii) data analysis including peptide/protein identification with the Mascot search algorithm and manual verification of an experimentally generated MS/MS spectrum with the theoretical MS/MS spectrum of identified fluorinated peptides. The experimental workflow for this proteomics approach is summarized in Figure 2, and we also described it previously [15].

Eighteen adult male Wistar rats (*Rattus norvegicus*) aged 10–16 weeks and with similar body weight were used in the study. Animals were provided with water and food ad libitum throughout the study period, and a 12 h day/night (12/12 h) rhythm, controlled temperature (21 ± 1 °C) and humidity (55 ± 5%) were maintained.

Rats were randomly assigned to the following groups: a control group (*n* = 6) and a group (*n* = 12) treated with cinacalcet at a dose corresponding to the maximum does used in humans. The second group was further divided into two subgroups: one treated for 7 days (*n* = 6) and the other for 21 days (*n* = 6). After the respective 7 and 21 days of experimental treatment, animals were sacrificed, and their tissues collected. Liver and brain tissue samples were used for these experiments. All tissues were deep-frozen immediately after collection and stored as such until measurements process starts.

### 5.1. Chemicals, Reagents, and Instrumentation

Analytical grade chemicals and analytical standards were obtained from Merck (Darmstadt, Germany), Promega (Madison, WI, USA), Thermo Scientific (Bartlesville, OK, USA), and EMD Millipore (Darmstadt, Germany). Deionized water from the Milli-Q system (18.2 MΩ cm; EMD Millipore, Darmstadt, Germany) was used for samples and standard dilution.

The instrumentation for extraction and sample preparation was as follows: mechanical homogenizer Ultra-Turrax (IKA, Königswinter, Germany), laboratory incubator CLN 240 (MultiSerw, Brzeźnica, Poland), vacuum centrifuge 5804/5804 R (Eppendorf, Enfield, CT, USA), vortex shaker (IKA, Königswinter, Germany), thermomixer Eppendorf Comfort (Eppendorf, Enfield, CT, USA), and vacuum concentrator SpeedVac Concentrator Plus (Eppendorf, Enfield, CT, USA). Reversed-phase capillary nano-UHPLC separations were performed using an UltiMate 3000 nano system (Dionex Ultimate Series UHPLC, Thermo Scientific, Bartlesville, OK, USA) equipped with in-house–packed capillary C-18 column (75 μm × 500 mm, particle size 1.9 μm) coupled on-line with a high-resolution tandem mass spectrometer (Orbitrap Fusion Tribrid™ Mass Spectrometer, Thermo Scientific, Bartlesville, OK, USA).

### 5.2. Sample Preparation and Liquid Chromatography–MS/MS Analysis

Before aby sample preparation, all tissues were unfrozen. Approximately 50 mg of tissues were exposed to with 1 mL of lysis buffer (1% SDS and cOmplete™ EDTA-free Protease Inhibitor in 100 mmol/L ammonium bicarbonate) at room temperature over 15 min, supported by a mechanically operated homogenizer. The supernatant was separated from the residue by centrifugation for 30 min at 20,000× *g*. Total protein concentration was determined using the Pierce™ BCA Protein Assay Kit (Thermo Scientific, Bartlesville, OK, USA) by following the manufacturer’s procedure. Volumes of derived protein extracts equivalent to 100 μg protein were precipitated overnight with acetone (−20 °C, 800 μL) and centrifuged (20,000× *g*) at 4 °C, and the supernatants were discarded. Protein pellets were then resuspended in 50 µL of 0.1% RapiGest in 50 mmol/L ammonium bicarbonate buffer. Proteins were reduced with 75 μL of 5 mmol/L 1,4-dithiothreitol for 45 min at 56 °C, then alkylated with 75 μL of 30 mmol/L acrylamide for 30 min. Alkylation was quenched by addition of an equimolar amount of 1,4-dithiothreitol and incubation at room temperature for 15 min. Samples were enzymatically digested for 18 h at 37 °C with 75 μL of 20 ng/L trypsin and 100 μL of 50 mmol/L ammonium bicarbonate (100:1 protein:enzyme weight ratio). The reaction was quenched by the addition of 150 μL 5% formic acid. Pierce™ Peptide Desalting Spin Columns (Thermo Scientific, Bartlesville, OK, USA) were used to purify peptides. Desalted peptides were vacuum dried at room temperature and re-suspended in 100 μL of 5% acetonitrile and 0.1% formic acid and analyzed. Liquid chromatography–tandem MS analysis was performed using an Orbitrap Fusion™ Tribrid™ mass spectrometer (Thermo Fisher Scientific, Bartlesville, OK, USA) coupled on-line to a nanoflow UHPLC instrument (Ultimate 3000 nanoUHPLC, Thermo Fisher Scientific, Bartlesville, OK, USA). One microgram of enzymatically cleaved peptides was separated on a reverse-phase 50-cm C18 in-house–packed column (75 μm inner diameter, ReproSil Gold 120 C18 1.9 μm beads; Dr. Maisch, Ammerbuch, Germany) in a reverse phase at a flow rate of 300 nL/min using gradient elution consisting of 0.1% formic acid in water (solvent A) and 0.1% formic acid in 80% acetonitrile/20% water (solvent B) for 360 min. The eluted compounds were ionized in positive ion mode with a capillary voltage of 1.9 kV in a nano-ESI ion source. The ion source parameters optimized on the total ion current values were as follows: auxiliary gas flow 15 L/min and ion transfer tube temperature 300 °C. Survey scans were recorded with the Orbitrap mass analyzer at a resolving power of 60,000 in the m/z range of 350–1600, and from each survey scan, the most abundant multiply charged ions were fragmented by higher energy collisional dissociation. The product ions were analyzed in the Orbitrap mass analyzer at a resolving power of 15,000. Cycle time was 3 s. After fragmentation, the masses were excluded for 30 s from further fragmentation.

### 5.3. Data Analysis

The raw MS data sets were processed to generate a peak list in Mascot generic format (*.mgf) using ThermoRawFileParser (v. 1.5.1) software (Thermo Scientific, Bartlesville, OK, USA). The peak lists were searched using the Mascot search engine (Matrix Science, London, UK, version: 2.4.1). Fluorination of alanine (A), phenylalanine (F), tryptophan (W), and tyrosine (Y) was set as variable modification. The fixed modification, derived from using acrylamide as an alkylating agent, was propionamidation on cysteine (C). Charge states of +2, +3, and +4 were considered for parent ions. Mass tolerance was set to ±10.0 ppm for parent ion masses and ±0.6 Da for fragment ion masses. The SwissProt database (accessed date: December 2020) was searched, with the specified taxonomy Rattus Norvegicus (Taxonomy ID: 10116). Data were filtered with a false discovery rate of 1% at the peptide level. Proteins and peptides were identified using a target–decoy approach with a reversed database. Only high confidence identifications of proteins were analyzed with a peptide ion score cut-off of 30 (consistent with a 1% chance of a false-positive match).

## 6. Conclusions

We showed that administration of fluorinated drugs results in tissue-specific fluorination of selected proteins in rats. To investigate this phenomenon after the administration of other drugs and in regard to other tissues further research should be performed.

## Figures and Tables

**Table 1:** List of confirmed identified fluorine-containing proteins based on automatic identification in the 21 day groups using Mascot software after manual verification.

Examined Tissue	Peptide Sequence	Variable Modification	Experimental Mass	Theoretical Mass	Δ Mass [ppm]	Peptide Score	Protein Name	Gene Name
Liver	G**A**SIPQFTNSPTMVIMVGLPAR	fluorination (A)	2304.1958	2304.1770	8.15	120	6-phosphofructo-2-kinase/ fructose-2,6-bisphosphatase 1 (Fragment)	*Pfkfb1*
	VIESTQDLGNDLAGVL**A**LQR	fluorination (A)	2129.0930	2129.1128	−9.29	112	Spectrin beta chain	*Sptbn2*
	LFATEATSDWLN**A**NNVPATPV AWPSQEGQNPSLSSIR	fluorination (A)	3985.9498	3985.9246	6.31	79	Carbamoyl-phosphate synthase [ammonia], mitochondrial	*Cps1*
Brain	GVVV**F**GEPITASLGTDGSHYWSK	fluorination (F)	2424.1777	2424.1762	0.61	73	Dihydropyrimidinase-related protein 4 (Fragment)	*Dpysl4*
	DLDLLNP**A**AR	fluorination (A)	1114.5835	1114.5782	4.49	47	Prominin-2	*Prom2*
	VIESTQDLGNDLAGVL**A**LQR	fluorination (A)	2129.1112	2129.1128	−0.78	43	Spectrin beta chain	*Sptbn2*
	ALSY**A**LR	fluorination (A)	810.4402	810.4399	0.37	36	tRNA phosphotransferase 1	*Trpt1*

The position of fluorination of al-anine (A) and fluorination of phenylalanine (F) were indicated in bold red.

## Data Availability

The authors confirm that the data supporting the findings of this study are available within the article and its Supplementary Material. Raw data that support the findings of this study are available from the first author, upon reasonable request.

## Supplementary Materials

The following supporting information can be downloaded at: https://www.mdpi.com/article/10.3390/ijms23084202/s1.
